# Supplementary material for: Bias-Modulated High Photoelectric Response of Graphene-Nanocrystallite Embedded Carbon Film Coated on n-Silicon
Source: Nanomaterials (Basel). 2019 Mar 1;9(3):327. doi: 10.3390/nano9030327 (PMC6473602; doi:10.3390/nano9030327)
Supplement: Supplementary file 1 [file nanomaterials-09-00327-s001.pdf]

Supplementary Materials:

Bias-Modulated High Photoelectric Response of Graphene-Nanocrystallite Embedded Carbon Film Coated on n-Silicon

Xi Zhang, Zezhou Lin, Da Peng and Dongfeng Diao*

Institute of Nanosurface Science and Engineering, Guangdong Provincial Key Laboratory of Micro/Nano Optomechatronics Engineering, Shenzhen University, Shenzhen, 518060, China; zh0005xi@szu.edu.cn (X.Z.), zezhoulin@163.com (Z.L.), pandavmac@163.com (D.P.)

* Correspondence: dfdiao@szu.edu.cn; Tel.: 86-755-26902415

1. FIB processing to observe the cross-section of GNEC film

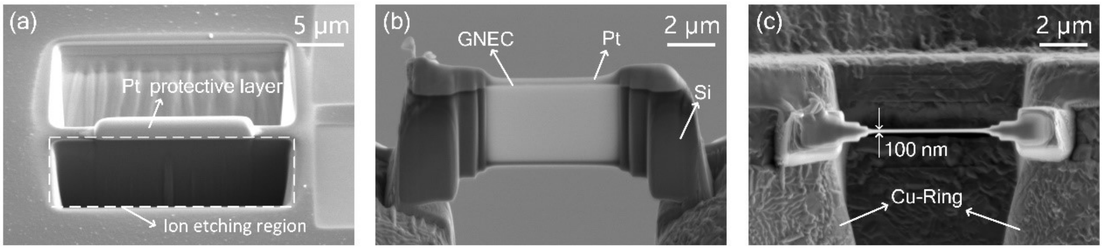

Figure S1. SEM images of (a) platinum protective layer evaporation and ion etching separation of cross-section, (b) a cross-section sample fabricated by using FIB, and sample located on a copper ring in preparation for TEM observation. (c) Top view of the cross-section, the thickness was thinned to 100 nm by ion irradiation.

2. Current-voltage (I-V_{diode}) curves of carbon films/n-Si

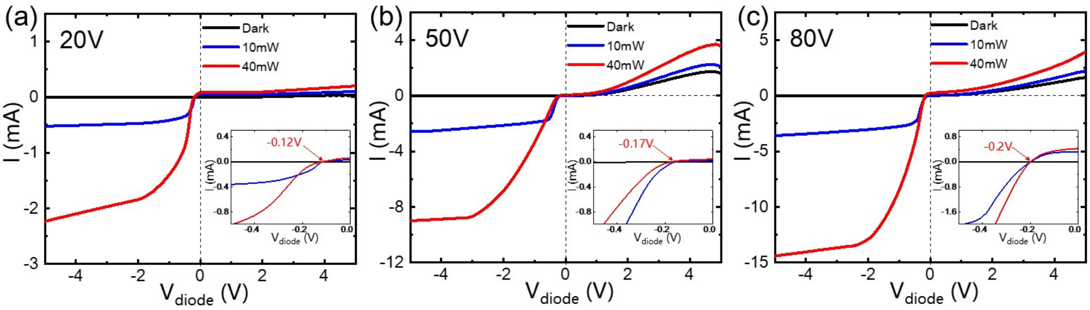

Figure S2. Current-voltage (I-V_{diode}) curves under darkness and illumination (P=10 mW or 40 mW) of 785 nm laser of carbon films/n-Si of V_{dep} at (a) 20V, (b) 50V, and (c) 80V. The GNEC film/n-Si exhibits a large photocurrent under reverse V_{diode}. As the reverse bias voltage increases from 0V to -2V~-3V, the photocurrent tends to saturate.
